# Identification of a Novel Mutation in *SASH1* Gene in a Chinese Family With Dyschromatosis Universalis Hereditaria and Genotype-Phenotype Correlation Analysis

**DOI:** 10.3389/fgene.2020.00841

**Published:** 2020-08-04

**Authors:** Nan Wu, Lili Tang, Xiuxiu Li, Yuwei Dai, Xiaodong Zheng, Min Gao, Peiguang Wang

**Affiliations:** ^1^Department of Dermatology, The First Affiliated Hospital, Anhui Medical University, Hefei, China; ^2^Institute of Dermatology, Anhui Medical University, Hefei, China; ^3^Key Laboratory of Dermatology, Anhui Medical University, Ministry of Education, Hefei, China; ^4^Provincial Laboratory of Inflammatory and Immune Mediated Diseases, Hefei, China

**Keywords:** dyschromatosis universalis hereditaria, *SASH1* gene, *ABCB6* gene, mutation, phenotype

## Abstract

Dyschromatosis universalis hereditaria (DUH) is a rare genodermatosis characterized by mottled hyperpigmented and hypopigmented macules. *SASH1* and *ABCB6* have been identified as the causative genes for this disorder. We performed whole exome sequencing on a Chinese family with DUH and genotype-phenotype correlation analysis in DUH and lentiginous phenotype patients. A novel heterozygous missense mutation p.Q518P in *SASH1* gene was detected in this family. A majority of patients with *SASH1* mutations presented as a distinct clinical phenotype clearly different from that in patients with *ABCB6* mutations. Our findings further enrich the reservoir of *SASH1* mutations in DUH. The clinical phenotypic difference between *SASH1* and *ABCB6* variants is suggestive of a close phenotype-genotype link in DUH.

## Introduction

Dyschromatosis universalis hereditaria (DUH) is a rare genodermatosis which is characterized by generalized hyperpigmented macules mixed with hypopigmented macules in a reticular pattern, which usually appears from infancy or early childhood. It was firstly described in 1933 by Ichikawa and Hiraga. DUH shares a typical phenotypic feature with dyschromatosis symmetrica hereditaria (DSH), that is reticulate hyperpigmentation and hypopigmentation on extremities. The mode of autosomal dominant inheritance was observed in approximately 50% of all cases. In addition, DUH was rarely accompanied with other abnormalities, such as neurosensory hearing loss, adermatoglyphia, photosensitivity, primary ovarian failure, insulin-dependent diabetes, renal failure, and ocular abnormalities, Dowling-Degos disease (DDD) (Yang and Wong, 1991; Sandhu et al., 2004; Bhoyar et al., 2015; Rojhirunsakool and Vachiramon, 2015; Gupta, 2016; Jayanthi et al., 2016).

DUH consists of three major types, namely DUH1 (OMIM 127500), DUH2 (OMIM 612715), and DUH3 (OMIM 615402) according to the chromosomal mapping of 6q24.2-q25.2, 12q21-q23, and 2q35 region respectively (Xing et al., 2003; Stuhrmann et al., 2008; Zhang et al., 2013). In 2013, Zhou et al. (2013) first identified three heterozygous missense mutations of the SAM (sterile alpha motif) and SH3 (Src homology domain 3) domain containing one (*SASH1*) gene in three unrelated families with DUH1, whereas, Zhang et al. (2013) identified three heterozygous missense mutations in *ABCB6* gene in a large family and six sporadic patients with DUH3. Thereafter, a number of mutations in *SASH1* and *ABCB6* were successively detected in other affected individuals with DUH (Cui et al., 2013; Liu et al., 2014; Lu et al., 2014; Zhong et al., 2019a, b). We herein described a case of Chinese DUH family with a novel missense mutation in *SASH1* gene, and performed genotype-phenotype correlation analysis in the DUH and lentiginous type patients from the published literatures.

## Case Presentation

**FIGURE 1:**
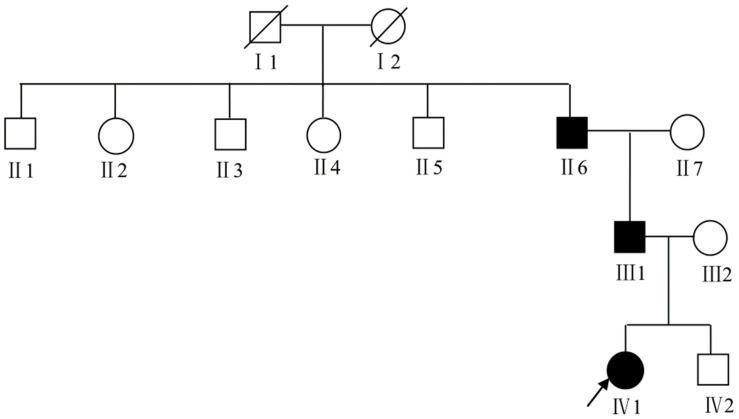
The pedigree chart of DUH family. The proband was marked with an arrow. Females were indicated by circles while males were indicated by squares. Blackened symbols represented patients who were carried the mutation through mutation sequencing.

**FIGURE 2:**
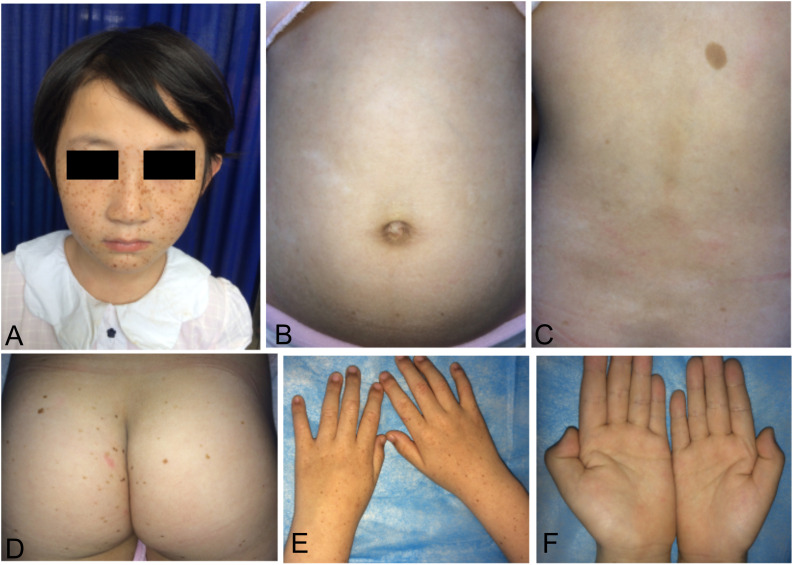
Cutaneous manifestations of the proband. **(A,D,E)** Light brown to dark brown macules on the her face, buttocks, and dorsa of hands. **(B,C)** Irregular hypopigmented macules on the her abdomen and back. **(F)** None of involvement on her palms.

**FIGURE 3:**
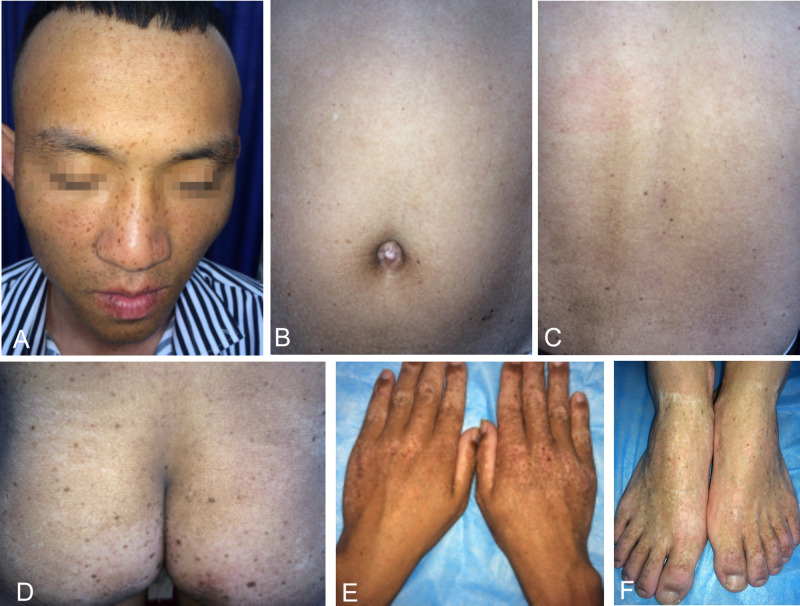
Cutaneous manifestations of the proband’s father. **(A–D)** Light brown to dark brown macules on his face, abdomen, back and buttocks, more prominent on his face. **(B–D)** Irregular hypopigmented macules or patches on his abdomen, back and buttocks. **(E,F)** Reticular dyschromatosis on the dorsa of his hands and feet.

We recruited a four-generation Chinese family with generalized dyschromatosis from Anhui Province in China. The pedigree is suggestive of an autosomal dominant inheritance (Figure 1). The proband was a 6-year-old girl who presented as asymptomatic hyperpigmented macules for 4 years. The pigmented lesions initially appeared on her cheek, and gradually spread to her entire body over a period of 3 years. On dermatological examination, light brown to dark brown macules in 2–4 mm of diameter were diffusely distributed on her face, dorsa of hands and buttocks, while only a small number of similar lesions scattered on her neck, trunk and limbs. Moreover, some irregular hypopigmented macules were also observed on her abdomen and back. Her palms, soles and oral mucosa were spared (Figure 2). Her general health seemed like to be normal. Her father and grandfather showed similar hyperpigmented macules on their whole body. However, obviously reticular hypopigmented macules or patches were also present on their buttocks as well as dorsa of their hands and feet. The hyperpigmentation, hypopigmentation and normal color of skin mingled together and constituted a characteristically variegated appearance on their extremities (Figure 3). The diagnosis of DUH was established by virtue of clinical features of all affected individuals in the family. Her grandmother, mother and younger brother were all normal.

## Methods

### Peripheral Blood Collection and DNA Extraction

After obtaining informed consent from all participants and approval from Clinical Research Ethics Committee of Anhui Medical University, EDTA anticoagulated venous blood samples were collected from three affected and three unaffected members for DNA analysis. Genomic DNA was extracted using the DNA extraction kit (Promega, Madison, WI, United States) in a standard procedure and stored at −80°C.

### Whole Exome Sequencing (WES)

The proband (IV1) and her father (III1) were selected to conduct the WES. Genomic DNA fragments corresponding to all exons in genome were amplified by polymerase chain reaction and subjected to automatic DNA sequencing after purification. Agilent SureSelect XT Library Prep Kit and SureSelect Human All Exon V6 kit were used for the library preparation and capture, followed by 150 bp pair-end sequencing on Illumina Hiseq XTen platform according to the manufacture’s recommendations. After quality control of the raw data, variants comparisons, mutation recognition and annotation, a list of suspected variants were obtained. Screening for disease-associated deleterious mutations was then made with emphases on all the possible pathogenic variations in reported *SASH1* and *ABCB6* genes, according to the frequency in the control database, variation types, and prediction of mutation function such as the score in SIFT and PolyPhen2 database.

### Sanger Sequencing

The possible pathogenic variations identified by WES were confirmed by Sanger sequencing in the proband’s grandparents (II6 and II7), mother (III2), and younger brother (IV2) to detect genotype-phenotype co-segregation. Primers flanking all coding regions of the possible variations in *SASH1* or *ABCB6* were designed using software Primer Premier 5.0 (Primer Biosystems, Foster City, CA, United States). PCR products from genomic DNA were sequenced using an ABI 3730XL DNA Analyzer (ABI, Foster City, CA, United States). The sequencing results were analyzed using Finch TV (Version 1.5), and the newly discovered mutation was named referring to the principle of the Human Genome Variation Society (HGVs).^1^

## Results

### WES Results and Co-segregation Analysis

**FIGURE 4:**
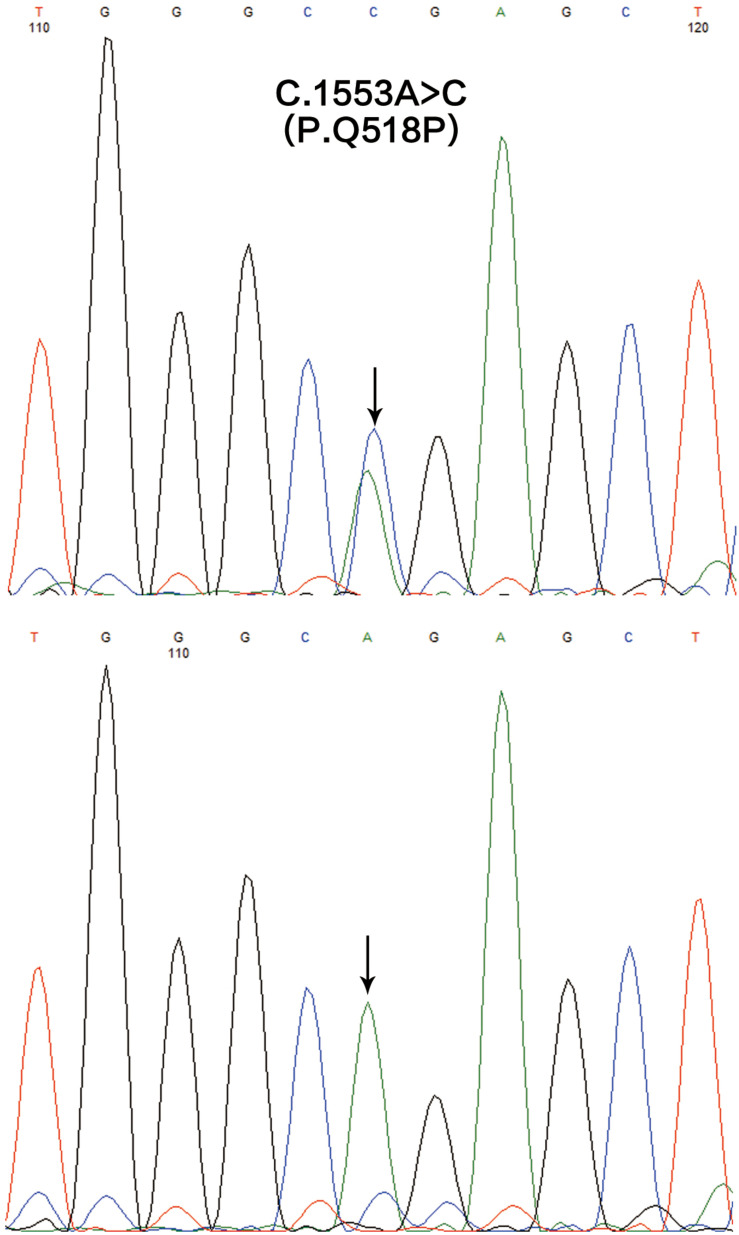
The mutation analysis of *SASH1* gene in this family. A heterozygous missense mutation c.1553A > C (p.Q518P) in *SASH1* identified in the affected individuals (the upper) and the reference sequencing of *SASH1* in the unaffected individuals (the lower).

We generated approximately 14.38 and 12.97 Gb of sequence data in the two affected (III1 and IV1) respectively. A heterozygous missense variation in *SASH1* gene (NM_015278.3), c.1553A > C, was identified after integrative analysis. This variation was not present in 700 normal controls. Sanger sequencing revealed that this variation was detected in the proband’s grandfather, but not present in three unaffected family members (Figure 4). The sequences of primers used for validation sequencing were *SASH1*-Forward primer: AAACCCATGGCAGGACTCGGG, *SASH1*-Reverse primer: TAGCACCTGAAGGGCAGGACTTG. This variation resulted in an amino acid substitution, p.Gln518Pro (p.Q518P), which co-segregated perfectly with the disease. Moreover, the substitution p.Q518P was predicted to be “deleterious” with a score of 0.001 in SIFT^2^ and “probably damaging” with a score of 0.999 in PolyPhen2.^3^ None of possible pathogenic variations was present in *ABCB6* gene.

### Genotype-Phenotype Correlation Analysis

So far, totally 11 heterozygous missense mutations in *SASH1* have been identified in patients with dyschromatosis (Table 1), in which seven mutations contribute to DUH phenotype and four mutations lead to multiple lentiginous. Comparing the phenotype of *SASH1* variants with that of *ABCB6* variants in DUH, we found a distinct difference in clinical phenotype between them. The most prominent feature in DUH patients with *SASH1* variants was generalized light brown to dark brown macules similar to leopard’s spots, especially on the sun-exposed areas, which may lead to the misdiagnosis as multiple lentigines for those DUH patients with multiple lentigines arising on the background of light-complexioned skin. However, the coexistence of variable retiform or homogenous hypopigmentation helps to differentiate multiple lentigines with DUH. The characteristic manifestation of *ABCB6* variants is typically generalized mottled hyperpigmented and hypopigmented macules arranging in a reticular pattern.

**TABLE 1:** Clinical and genetic information in families with DUH or lentiginous phenotype.

**Number**	**Clinical phenotypes**	**Onset age**	**Inheritance pattern**	**Gene**	**Mutation type**	**Exon**	**Nucleotide change**	**Amino Acid change**	**References**
1	DUH	Unknown	AD	*SASH1*	Heterozygous	13	c.2000G > A	p.Glu509Lys	Zhou et al., 2013
2	DUH	Unknown	AD	*SASH1*	Heterozygous	13	c.2019T > C	p.Leu515Pro	Zhou et al., 2013
3	DUH	Unknown	AD	*SASH1*	Heterozygous	14	c.2126T > G	p.Tyr551Asp	Zhou et al., 2013
4	DUH	3 years	AD	*SASH1*	Heterozygous	15	c.1784T > C	p.Met595Thr	Zhong et al., 2019b
5	DUH	7 months	AD	*SASH1*	Heterozygous	14	c.1651T > C	p.Tyr551His	Zhong et al., 2019b
6	DUH	<1 year	AD	*SASH1*	Heterozygous	15	c.1761C > G	p.Ser587Arg	Our previous study^a^
7	DUH	12 years	AD	*SASH1*	Heterozygous	15	c.1761C > G	p.Ser587Arg	Cui et al., 2020
8	DUH	2 years	AD	*SASH1*	Heterozygous	15	c.1553A > C	p.Gln518Pro	Our present study^a^
9	Lentiginous phenotype	1 year	AD	*SASH1*	Heterozygous	13	c.1556G > A	p.Ser519Asn	Shellman et al., 2015
10	Lentiginous phenotype	3 years	AD	*SASH1*	Heterozygous	13	c.1537A > C	p.Ser513Arg	Zhang et al., 2016
11	Lentiginous phenotype	Unknown	sporadic	*SASH1*	Heterozygous	13	c.1527_1530dupAAGT	p.Leu511Lysfs^∗^21	Zhang et al., 2016
12	Lentiginous phenotype	18 months	unknown	*SASH1*	Heterozygous	13	c.1519T > G	p.Ser507Ala	Wang et al., 2017
13	DUH-like pigmentation and skin carcinoma	1 year	AR	*SASH1*	Homozygous	15	c.1849G > A	p.Glu617Lys	Courcet et al., 2015
14	DUH	2 years	AD	*ABCB6*	Heterozygous	3	c.1067T > C	p.Leu356Pro	Zhang et al., 2013
15	DUH	Unknown	sporadic	*ABCB6*	heterozygous	1	c.508A > G	p.Ser170Gly	Zhang et al., 2013
16	DUH	Unknown	sporadic	*ABCB6*	Heterozygous	12	c.1736G > A	p.Gly579Glu	Zhang et al., 2013
17	DUH	2 months	AD	*ABCB6*	Heterozygous	11	c. 1663C > A	p.Gln555Lys	Cui et al., 2013
18	DUH	Unknown	sporadic	*ABCB6*	Heterozygous	1	c.459delC	p.Trp154Glyfs^∗^96	Cui et al., 2013
19	DUH	Unknown	AD	*ABCB6*	Heterozygous	7	c.1358C > T	p.Ala453Val	Liu et al., 2014
20	DUH	Unknown	sporadic	*ABCB6*	Heterozygous	7	c.1358C > T	P.Ala453Val	Liu et al., 2014
21	DUH	Unknown	AD	*ABCB6*	Heterozygous	4	c.964A > C	p.Ser322Lys	Liu et al., 2014
22	DUH	<1 year	AD	*ABCB6*	Heterozygous	4	c.964A > C	p.Ser322Lys	Lu et al., 2014
23	DUH	<1 year	unknown	*ABCB6*	Heterozygous	6	c.1270T > C	p.Tyr424His	Lu et al., 2014
24	DUH	2 years	AD	*ABCB6*^*b*^	Heterozygous	6	c.1270T > C	p.Tyr424His	Liu et al., 2016
25	DUH	12 years	AD	*ABCB6*	Heterozygous	15	c.2017A > G	p.Thr673Ala	Zhong et al., 2019a
26	XP-C with DUH-like dyschromatosis	15 months	unknown	*ABCB6*^c^	Heterozygous	8	c.1400A > G	p.Asn467Ser	Masaki et al., 2018

*^*a*^Our previous study published in the Chinese Journal of Dermatology in 2016; ^*b*^With a heterozygous nonsense mutation in ADAR1 gene (c. 1325C > G) and a heterozygous mutation in ABCB6 gene; ^*c*^With a homozygous nonsense mutation (c.1735C > T, p.R579X) in XPC gene and a heterozygous mutation in ABCB6 gene.*

## Discussion

**FIGURE 5:**
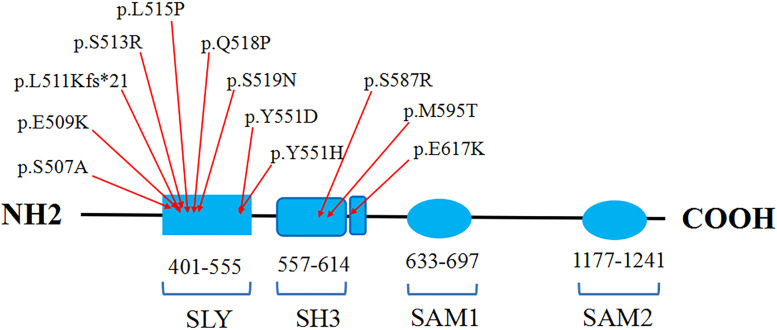
The schematic representation of the protein domains and the amino acid positions of *SASH1* mutations responsible for DUH and lentiginous phenotype.

*SASH1* was firstly identified as a potential tumor suppressor gene in breast cancer, which played a crucial role in tumorigenesis, development, invasion and metastasis (Zeller et al., 2003; Lin et al., 2012). In addition, *SASH1* was also involved in various complex signaling pathways as a pivotal regulator or mediator (Dauphinee et al., 2013). Zhou et al. (2013) found Gαs-SASH1-IQGAP1-E-Cadherin signaling pathway was responsible for melanocytes transepithelial migration in the progression of DUH. Lately, Zhou et al. (2017) demonstrated that *SASH1* mediates skin melanogenesis through a cascade of p53/α-MSH/POMC/Gαs/SASH1. So far, totally 11 heterozygous missense mutations in *SASH1* have been identified in some patients with dyschromatosis as illustrated in the Table 1, including nine mutations (p.Y551H, p.S507A, p.S513R, p.L511Kfs^∗^21, p.S519N, p.Y551D, p.L515P, p.E509K, p.Q518P) in SLY domain and two mutations (p.S587R, p.M595T) in SH3 domain, in which seven mutations contribute to DUH phenotype and four mutations lead to multiple lentiginous phenotype (Shellman et al., 2015; Zhang et al., 2016; Wang et al., 2017). Besides, a homozygous missense mutation p.E617K in *SASH1* was responsible for an autosomal recessive syndrome featuring DUH-like pigmentation anomaly, palmoplantar keratoderma and skin carcinoma (Courcet et al., 2015). The protein domains and the amino acid positions of SASH1 mutations responsible for DUH and lentiginous phenotype were showed in a schematic representation (Figure 5). In this study, we detected a missense mutation (p.Q518P) in *SASH1* gene, which is located in the highly conserved SLY domain of SASH1. Considering the observed heterogeneity of DUH phenotypes in this family, we wondered whether it was related to *SASH1* heterozygous variant coupled with variants in other regions, such as 12q21-q23 which is associated with DUH2. Hence, we explored the variants in this region from the WES data, however, we did not find any pathogenic variation in this region, suggesting that the heterogeneity in this family was related to *SASH1* variation.

Previously we have identified a heterozygous missense mutation (p.S587R) in *SASH1* in a large DUH family from Henan Province, as reported by Yao et al. in *Chin J Dermatol* in 2016. All seven affected individuals in the family had extensive light brown to dark brown macules with 2–5 mm in diameter on their whole body. Moreover, homogenous hypopigmentation on the rest of skin occurred in six affected individuals. Only one affected individual manifested as generalized reticulate hypopigmented macules. Most recently, this missense mutation (c.1761C > G, p.S587R) has also been identified in a large Chinese family with four generation (Cui et al., 2020). Zhong et al. (2019a) described two unrelated DUH families carrying mutations in *SASH1*. The probands in these two families both presented with generalized light to dark brown macules on their face, neck, trunk and limbs. In addition, reticular hypopigmentation was also seen on their neck as well as dorsal aspects of hands and feet. Apparently, there is a significant phenotypic heterogeneity among intra-family and inter-family members with DUH in the light of these cases mentioned above.

Besides DUH, Shellman et al. (2015) identified a missense mutation in *SASH1* in an autosomal dominant lentiginous phenotype. All affected members exhibited generalized dark brown macules, more prominent in sun-exposed areas. Moreover, some reticular hypopigmented macules were also present on the dorsa of an affected member’s hands. Zhang et al. (2016) detected two mutations in *SASH1* in two cases of patients with lentiginous phenotype. These two patients both presented with generalized light brown to dark brown macules, particularly on their face and neck. Furthermore, some scattered hypopigmented spots were also observed on the trunk and face and mild dyschromatosis on the elbow and dorsal area of hands and feet in one patient, while diffuse hypopigmentation occurred on his entire body in the other patient. Later, Wang et al. (2017) reported that a family manifesting as multiple lentigines carried a mutation in *SASH1*. The proband showed substantially identical phenotype as an affected individual in a DUH family with a mutation (p.S587R) in *SASH1*. Intriguingly, all affected individuals with lentiginous phenotype shared very similar clinical features with the DUH patients. More importantly, the development of their pigmented defects was associated with a mutation in *SASH1*. It is well-known that multiple lentigines related to Noonan syndrome or LEOPARD syndrome is caused by mutations in *PTPN11* (Tartaglia et al., 2001; Digilio et al., 2002). Hence, we consider that these cases with lentiginous phenotype are actually DUH because of high similarity in their clinical phenotype and molecular genetics.

Three mutations (p.S170G, p.L356P, p.G579E) in *ABCB6* were determined in a large Chinese family and six sporadic patients with DUH (Zhang et al., 2013). The proband from the family had generalized hyperpigmented and hypopigmented macules in a reticular arrangement with mottled appearance. Thereafter, six mutations (p.S322K, pA453V, p.Y424H, p.T673A, p.Q555K, c. p.W154Gfs^∗^96) in *ABCB6* were detected in eight families and five sporadic cases with DUH (Cui et al., 2013; Liu et al., 2014; Lu et al., 2014; Zhong et al., 2019a). Besides, a case of xeroderma pigmentosum (XP) complementation group C with DUH-like pigmentation was reported, and a heterozygous missense mutation (p.N467S) was found (Masaki et al., 2018). All affected individuals also exhibited generalized variegated hyperpigmented and hypopigmented macules in a reticulate arrangement over their entire body or abdomen and dorsa of hands. Comparing the phenotype of *SASH1* variants with that of *ABCB6* variants in DUH, we find a distinct difference in clinical phenotype between them. The most prominent feature of *SASH1* variants in DUH is generalized light brown to dark brown macules similar to leopard’s spots, especially on the sun-exposed areas. Therefore, if not carefully examined, they are easily misdiagnosed as multiple lentigines for those DUH patients with multiple lentigines arising on the background of light-complexioned skin. However, the coexistence of variable retiform or homogenous hypopigmentation helps to differentiate multiple lentigines with DUH. The characteristic manifestation of *ABCB6* variants is typically generalized mottled hyperpigmented and hypopigmented macules arranging in a reticular pattern. In contrast, it is not difficult to make a correct clinical diagnosis for the DUH cases associated with mutations in *ABCB6*. Clinically, the DUH case is rare, so herein we only describe a case of DUH family with a novel mutation in *SASH1* gene. The analysis of genotype-phenotype correlation in the patients with DUH or lentiginous phenotype were mainly dependent on the information on the published literatures.

In summary, we successfully identified a novel pathogenic mutation in *SASH1* in a Chinese family with DUH, thus further extending the mutation spectrum of this gene. The clinical phenotypic difference between *SASH1* and *ABCB6* variants is suggestive of a close phenotype-genotype link in DUH. Our findings not only help to enhance more comprehensive understanding for DUH, but also provide a useful clue for the correct diagnosis of DUH.

## Data Availability Statement

The datasets for this article are not publicly available due to concerns regarding participant/patient anonymity. Requests to access the datasets should be directed to the corresponding authors.

## Ethics Statement

Written informed consent was obtained from the individuals or the legal guardian for the publication of any potentially identifiable images or data included in this article.

## Author Contributions

NW conducted Sanger sequencing and wrote the manuscript. XL and YD collected clinical data and blood samples, and performed DNA extraction. LT performed whole exome sequencing and wrote the manuscript. XZ performed data analysis of the whole exome sequencing. MG and PW were responsible for the study design and guiding of the study implementation, and revised the manuscript. All authors contributed to the article and approved the submitted version.

## Conflict of Interest

The authors declare that the research was conducted in the absence of any commercial or financial relationships that could be construed as a potential conflict of interest.

## Footnotes

1 http://www.hgvs.org/

2 http://sift.bii.a-star.edu.sg

3 http://genetic.bwh.harvard.edu/pph2
